# Comparative Transcriptome Analysis between Broccoli (*Brassica oleracea var. italica*) and Wild Cabbage (*Brassica macrocarpa* Guss.) in Response to *Plasmodiophora brassicae* during Different Infection Stages

**DOI:** 10.3389/fpls.2016.01929

**Published:** 2016-12-23

**Authors:** Xiaoli Zhang, Yumei Liu, Zhiyuan Fang, Zhansheng Li, Limei Yang, Mu Zhuang, Yangyong Zhang, Honghao Lv

**Affiliations:** Group of Cabbage and Broccoli Breeding, Institute of Vegetables and Flowers – Chinese Academy of Agricultural SciencesBeijing, China

**Keywords:** clubroot, *Plasmodiophora brassicae*, broccoli, wild cabbage, RNA sequencing

## Abstract

Clubroot, one of the most devastating diseases to the Brassicaceae family, is caused by the obligate biotrophic pathogen *Plasmodiophora brassicae*. However, studies of the molecular basis of disease resistance are still poor especially in quantitative resistance. In the present paper, two previously identified genotypes, a clubroot-resistant genotype (wild cabbage, B2013) and a clubroot-susceptible genotype (broccoli, 90196) were inoculated by *P. brassicae* for 0 (T0), 7 (T7), and 14 (T14) day after inoculation (DAI). Gene expression pattern analysis suggested that response changes in transcript level of two genotypes under *P. brassicae* infection were mainly activated at the primary stage (T7). Based on the results of DEGs functional enrichments from two infection stages, genes associated with cell wall biosynthesis, glucosinolate biosynthesis, and plant hormone signal transduction showed down-regulated at T14 compared to T7, indicating that defense responses to *P. brassicae* were induced earlier, and related pathways were repressed at T14. In addition, the genes related to NBS-LRR proteins, SA signal transduction, cell wall and phytoalexins biosynthesis, chitinase, Ca^2+^ signals and RBOH proteins were mainly up-regulated in B2013 by comparing those of 90196, indicating the pathways of response defense to clubroot were activated in the resistant genotype. This is the first report about comparative transcriptome analysis for broccoli and its wild relative during the different stages of *P. brassicae* infection and the results should be useful for molecular assisted screening and breeding of clubroot-resistant genotypes.

## Introduction

Clubroot disease, caused by the obligate biotrophic pathogen *Plasmodiophora brassicae*, is one of the most devastating diseases to the Brassicaceae family (Ludwig-Müller et al., 2009). For many years, this soil-borne disease has caused declines in both the quality and yields of cruciferous crops worldwide (Dixon, 2009). The life cycle of *P. brassicae* consists of two distinct stages: the primary stage and the secondary stage. During the primary stage (root–hair infection stage), motile zoospores attach to the surface of root hairs, encyst, and release an amoeboid unit into the cell to form primary plasmodia. The plasmodia undergo a series of cell divisions to form multinucleate secondary plasmodia, which induce cell hypertrophy and cell hyperplasia in the tissues of the cortex and stele, leading to the development of galls. Eventually, the secondary plasmodia cleave into numerous resting spores that are released into the soil when the galls disintegrate (Rolfe et al., 2016). These *P. brassicae* spores can survive in the soil for up to 20 years, making the disease difficult to control in the field once soil is contaminated (Kageyama and Asano, 2009). Thus, studies on strategies and technologies for reducing the risk of clubroot contamination of cruciferous plants, especially cultivated crops, are urgently needed.

Screening and breeding clubroot-resistant (CR) cultivars is an effective and economical approach for reducing the use of chemical fungicides while minimizing crop losses. Some CR cultivars of cruciferous crops such as Chinese cabbage, cabbage, cauliflower, and oilseed rape are available for commercial production, but resistance may be overcome by rapidly evolving pathogen populations after large-scale application (Diederichsen et al., 2009). Therefore, a better understanding of the molecular mechanisms of clubroot disease development and host–*P. brassicae* interactions may provide strategies for improving plant tolerance of *P. brassicae* infections and establishing a theoretical basis for breeding resistant varieties.

*Arabidopsis thaliana* can also be infected by *P. brassicae* and therefore serves as a useful model host for studying the disease (Koch et al., 1991; Mithen and Magrath, 1992; Ludwig-Müller et al., 2009). Previous studies have advanced our understanding of the events leading to clubroot formation using various methods, including resistance screening (Fuchs and Sacristan, 1996), mutant analysis (Siemens et al., 2002, 2008; Alix et al., 2007), and genetic analysis of resistance (Jubault et al., 2008b; Gravot et al., 2011). In the past decade, several“-omics” approaches have been employed to elucidate the dynamic changes in protein composition and gene expression related to metabolic pathways during clubroot disease and more complex signaling pathways that are activated in response to *P. brassicae* infection. Deckers (2006), who focused on changes in the protein compositions in *A. thaliana*, showed that proteins involved in metabolism, cell defense, cell differentiation, and detoxification were differentially regulated during the initial infection stage. Several studies have addressed the roles of genes related to metabolism, hormone signaling, and defense responses based on transcriptome analyses in specific developmental stages (Siemens et al., 2006; Jubault et al., 2008a; Ludwig-Müller et al., 2009; Agarwal et al., 2011; Schuller et al., 2014).

It is also clear that there are differences in the resistance response between *A. thaliana* and *Brassica* species (Kobelt et al., 2000). Thus, previous studies have focused on understanding the mechanisms underlying clubroot disease resistance in *Brassica* species, including *Brassica rapa* and *Brassica napus*. Chu et al. (2014) identified differentially expressed genes (DEGs) involved in the signaling metabolism of jasmonate and ethylene, defensive deposition of callose and the biosynthesis of indole-containing compounds, which were all significantly up-regulated in clubroot-resistant plants compared with susceptible cultivars. More recently, Chen et al. (2016) confirmed that genes associated with PAMPs, calcium ion influx, hormone signaling, PR, transcription factors, and cell-wall modification played important roles in host–*P. brassicae* interactions during the early stages of infection by *P. brassicae*, based on transcriptome analysis of *B. rapa*. Xu et al. (2016) provided evidence of the involvement of IAA pathway-related genes during the root–hair infection stage in the roots and leaves of *B. napus* soon after *P. brassicae* inoculation based on qRT-PCR analysis.

*Brassica oleracea* is one of the most important worldwide leafy vegetables. Clubroot results in severe losses of yield and quality in *B. oleracea* as well as in other *Brassica* crops. However, there is little information on the molecular mechanisms of clubroot resistance in *B. oleracea* and its wild relatives obtained through transcriptome analysis, especially on studies that compare responses to *P. brassicae* infection between susceptible and resistant plant lines. Besides, previous studies have showed that clubroot resistance in *B. oleracea* is quantitative under polygenic control (Tomita et al., 2013). Up until now, only a few studies, done on *Arabidopsis*, have focused on the mechanisms controlling quantitative resistance (Jubault et al., 2013). Hence, for molecular mechanisms of resistance in *B. oleracea*, it is very necessary and urgent to get a comprehensive understanding through transcriptome analysis.

Here, comparative transcriptome analysis was employed for the roots of two genotypes (broccoli and its wild relative) to clarify two major issues: (1) what are the differences in the transcriptional response to *P. brassicae* infection associated with different stages; and (2) what is the genetic basis for the differences in resistance to clubroot between the two genotypes? Through the bioinformatics analysis, we believe that our results would provide new insight into the molecular mechanisms that impart high resistance to clubroot.

**Table 1:** Evaluation of B2013 and 90196 responses to pathotype 4 of *Plasmodiophora Brassicae.*

Genotype	Disease index (DI)	Resistant/susceptible
B2013	94.05 ± 2.06	R
90196	10.71 ± 3.57	S

**FIGURE 1:**
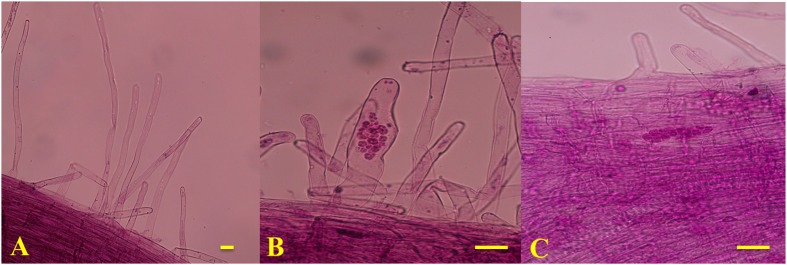
**Microscope observation results of T0 (A)**, T7 (**B**; the primary infection stage) and (**C**; the secondary infection stage). Bars = 1 μm.

## Materials and Methods

### Plant Material and Pathogen Isolate

On the basis of a previous study (Zhang, 2014), two identified genotypes—a clubroot-susceptible inbred broccoli line (90196, *B. oleracea var*. *italica*) and a clubroot-resistant wild cabbage line (B2013, *Brassica macrocarpa* Guss.) — were used in the present study. The wild type was able to intercross and produce fertile progeny with cultivated *B. oleracea* crops, including 90196. The root galls used in this study were collected from clubroot-infested field plots of Chinese cabbage in Tong’hai, Yunnan, China. The strain of the resting spores isolated from the galls was identified as pathotype 4 (Zhang, 2014), according to the differential classification of Williams (1966). Preparation of the resting spore suspension was performed as described by Luo et al. (2014), and the spore suspension was diluted with sterile water to 3 × 10^8^ spores/mL and stored at 4°C.

### *P. brassicae* Inoculation

To facilitate observation of the infection stage, infection of *P. brassicae* was performed in an improved culture solution according to Luo et al. (2014), with slight modification. Plant seeds were germinated on wet filter paper for 6 days (25°C, 16-h photoperiod). Then, 15-ml centrifuge tubes were wrapped in silver paper, and half-strength Hoagland’s nutrient solution (Hoagland and Arnon, 1950), which was used as the culture solution, was added to the tubes, after which the pH was adjusted to 6.0. One seedling was placed at top of each tube, held in place with a thin sponge. The plants were inoculated after 3 days of growth in solution by pipetting 1 mL of spore suspension (3 × 10^8^ spores/mL) into the tube; control plants were inoculated with an equivalent amount of sterile water. The seedlings were maintained for the duration of the experiment in an intelligent artificial climate chamber (RXZ, Jiangnan Instrument, Ningbo, China; 16-h light at 25°C day, 8-h dark at 20°C, 75% relative humidity). Fresh nutrient solution was added to the tubes as required.

### Microscopic Investigation and Tissue Sampling

To determine the timing of primary and secondary infection in the two genotypes, the infection processes of *P. brassicae* in the roots were magnified using an optical microscope (Olympus CX31, Japan) and imaged with a camera (Nikon 550D, Japan) every day after inoculation until the secondary infection was established. The spores were removed from the roots by gentle, thorough washing with sterile water. The roots were cut into 1-cm sections, which were then observed under a microscope after being fixed in formyl acid (FAA) and stained with modified carbol fuchsin. The stained root segments were mounted onto glass slides with a coverslip. At each time point, five individual plants were examined and three roots were observed from each plant.

Based on the observed infection stages, primary and secondary infection was found to occur at seven and 14 days after inoculation (DAI), respectively. Thus, the roots of 90196 and B2013 plants were sampled at 0 (T0), 7 (T7), and 14 (T14) DAI for the analysis of DEGs at different stages of infection. The roots of 10 (two biological replications, five plants for each replicate) plants were sampled and pooled for RNA extraction at each time point, for RNA sequencing. The roots were washed with sterile water, then immediately frozen in liquid nitrogen and stored at –80°C until use. To verify successful infection, plants were transplanted to nutrition pots (9 cm × 9 cm) containing a mixture of vermiculite: compost (1:2, v/v) after inoculation for 20 days in culture solution. The plants were gently removed from the soil at 42 DAI. The severity of symptoms was recorded using the scale previously described for *B. oleracea* (Manzanares-Dauleux et al., 2000), and the disease index (DI) was calculated as described by Manzanares-Dauleux et al. (2000). A line showing a DI value higher than 25 was considered to be susceptible to clubroot. The experiment was repeated three times.

### RNA Extraction, Sequencing, and *De novo* Assembly

Total RNA was extracted from a mixture of the root tissues of five individual plants as one replication for each of T0, T7, and T14 using the RN38-EASY SPIN Plus Plant Kit (BioTeke, Beijing, China), following the manufacturer’s instructions. The integrity of the RNA was verified through RNase-free agarose gel electrophoresis, and the concentration was measured using a 2100 Bioanalyzer (Agilent Technologies, Santa Clara, CA, USA). High-quality RNA from each replication of each treatment was pooled in equal quantities to generate one mixed sample for RNA sequencing. A cDNA library was constructed for each of the 12 mixed RNA samples and sequenced on the Illumina HiSeq 4000™ platform (Illumina, Inc., San Diego, CA, USA), which was conducted by the Allwegene Technology Company in Beijing, China. Before assembly, adapter sequences were removed from the raw reads. Low-quality reads (>50% bases with quality scores ≤5) and unknown bases (>10% N bases) were removed from each dataset to obtain more reliable results. The high-quality clean reads from all 12 samples were merged and assembled via the Trinity method (Grabherr et al., 2011) to construct unique consensus sequences for use as reference sequences.

### Analysis of Differential Gene Expression and Gene Annotation

The sequencing reads for each sample were remapped to the reference sequences using RSEM software (Li et al., 2011). Gene expression levels were measured using the FPKM (Fragments Per Kilobase of transcript per Million fragments) method (Trapnell et al., 2010) based on the number of uniquely mapped reads. For genes with more than one alternative transcript, the longest transcript was selected to calculate the FPKM. The DESeq package (ver. 2.1.0) (Anders and Huber, 2010) was employed to detect DEGs between two samples. The false discovery rate (FDR) was applied to correct the *p*-value threshold in multiple tests (Benjamini and Hochberg, 1995). An FDR-adjusted *p*-value (*q*-value) ≤ 0.05 and a |log2FoldChange| > 1 were used as the threshold for identifying significant differences in gene expression in this study.

All expressed genes were functionally annotated using five databases, including the NCBI non-redundant protein (Nr), Eukaryotic Ortholog Groups (KOG^1^), Protein family (Pfam^2^), Swiss-Prot^3^, and Kyoto Encyclopedia of Genes and Genomes (KEGG^4^) databases, employing BlastX (v. 2.2.28+) with an E-value of less than 1e-5. Gene Ontology (GO^5^) annotations were analyzed using Blast2GO (v.2.5) (Conesa et al., 2005). For a gene that was matched to multiple protein sequences, the protein with the highest similarity score was considered the optimal annotation. To infer the transcriptional changes over time in the two genotypes under *P. brassicae* infection, DEGs at 7 and 14 DAI were identified by comparing the expression levels at T7 with those at T0 and the level at T14 with those at T7 in B2013 and 90196, respectively. For convenience, DEGs showing higher expression levels at T7 than at T0 and those exhibiting higher expression at T14 than at T7 were designated “up-regulated,” whereas those that displayed lower expression were designated “down-regulated.”

### Gene Expression Validation

Eight genes with different expression levels, as revealed via RNA sequencing, were randomly selected for validation using quantitative real-time RT-PCR (qRT-PCR). The gene-specific primers designed according to the gene sequences using Primer Premier5 ^6^ were listed in Supplementary Table S1. Three technical replicates were performed for each gene. First-strand cDNA was synthesized using the PrimeScript RT reagent Kit (TAKARA BIO, Inc., Shiga, Japan). The relative transcription level of each gene was estimated in terms of threshold cycles using the 2^–ΔΔCT^ method (Livak and Schmittgen, 2001), and broccoli β-actin was employed as an internal control (Gómez-Lobato et al., 2012). qRT-PCR was carried out using SYBR Premix Ex TaqII (Tli RNaseH Plus; TAKARA BIO, Inc., Shiga, Japan) on an ABI Prism^®^ 7900HT (Applied Biosystems, Carlsbad, CA, USA) according to the manufacturer’s instructions.

## Results

### Disease Severity in the Two Genotypes and Infection Processes

The disease severity of the two genotypes was assessed at 42 DAI with *P. brassicae*. B2013 plants infected with pathotype 4 of *P. brassicae* showed only tiny clubs, with a mean DI of 10.71 at 42 DAI, while 90196 was susceptible in response to inoculation with the same *P. brassicae* pathotype, with a mean DI of 94.05, exhibiting a large club that replaced the main and secondary root systems (**Table 1**; **Supplementary Figure S1**). The infection process of *P. brassicae* between B2013 and 90196 was monitored after inoculation. At 7 and 14 DAI, microscopic observations revealed that root–hair infection and cortical infection were both present in B2013 and 90196 (**Figure 1**). For this reason, three time points (0, 7, 14 DAI) were selected to investigate the differential transcript changes between the two genotypes.

### RNA Sequencing and *De novo* Assembly of the Root Transcriptome of the Two Genotypes

Approximately 49.53-80.92 million 150-bp paired-end reads were generated from the 12 samples through RNA sequencing (**Table 2**). The GC content of the sequence data from the 12 libraries ranged from 46.20 to 50.50%, and the Q30% values (reads with an average quality scores > 30) were all ∼90%, indicating that the quality and accuracy of sequencing data were sufficient for further analysis. The percentage of sequenced reads from all libraries remapped to the assembled reference transcripts was ∼70%. After sequence trimming, the retained high-quality reads of all samples were merged and *de novo* assembled into 65,135 unigenes as reference transcripts, and 44,519 of these unigenes were functionally annotated in at least one database with an e-value cutoff of 1e-5. The N50 of the assembled genes was 1490 bp, with an average length of 915.5 bp and a maximum length of 14,801 bp. The sequencing data has been deposited into NCBI sequence read archive (SRA) under BioProject accession PRJNA345072 (alias: SRP090719).

**Table 2:** Sequencing and assembly statistics for the 12 transcriptome data of two genotypes at different stages during *P. brassicae* infection.

Samples ID		No. of raw reads ( × 10^6^)	No. of clean reads ( × 10^6^)	No. of base pairs ( × 10^9^)	GC content (%)	Q30 (%)	No. of mapped reads ( × 10^6^)	Mapped percentage (%)
B2013	14DAI_1	66.24	62.77	9.42	48.68	94.30	44.73	71.26
	14DAI_2	59.30	55.47	8.32	50.50	94.34	40.80	73.60
	7DAI_1	61.34	56.37	8.46	47.40	90.15	40.08	71.11
	7DAI_2	57.55	52.45	7.86	47.48	89.64	37.22	70.96
	0DAI_1	80.92	74.59	11.18	46.87	94.46	52.41	70.27
	0DAI_2	55.08	50.44	7.56	46.91	94.58	35.03	69.45
90196	14DAI_1	49.53	47.15	7.08	46.55	94.44	32.67	69.29
	14DAI_2	59.36	56.61	8.50	47.20	94.38	39.41	69.60
	7DAI_1	67.26	61.89	9.28	46.55	89.89	43.47	70.24
	7DAI_2	67.28	61.91	9.28	46.45	90.48	43.85	70.83
	0DAI_1	63.85	59.11	8.86	46.28	90.60	41.14	69.59
	0DAI_2	57.96	53.39	8.00	46.20	90.36	37.64	70.50

### RNA Sequencing Validation by qRT-PCR

**FIGURE 2:**
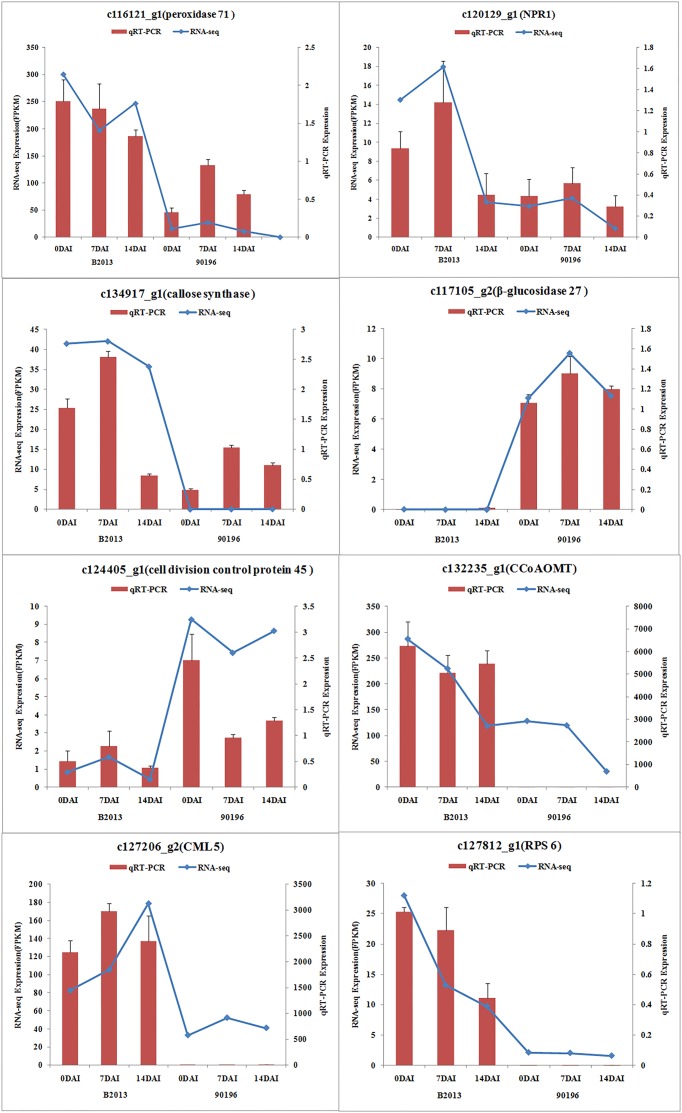
**Expression of the selected eight genes inferred by RNA -sequencing and qRT-PCR.** Data from qRT-PCR are means of two replicates and bars represent SE.

To validate the results obtained from RNA sequencing, eight genes with different expression levels were randomly selected to perform qRT-PCR. For each gene, the FPKM values of transcriptome data exhibited similar expression trends at all the three time stages comparing with the results of qRT-PCR (**Figure 2**). It suggested a reliable expression results generated by RNA sequencing.

### Gene Expression Pattern Analysis, Clustering, and Functional Enrichment of DEGs

**FIGURE 3:**
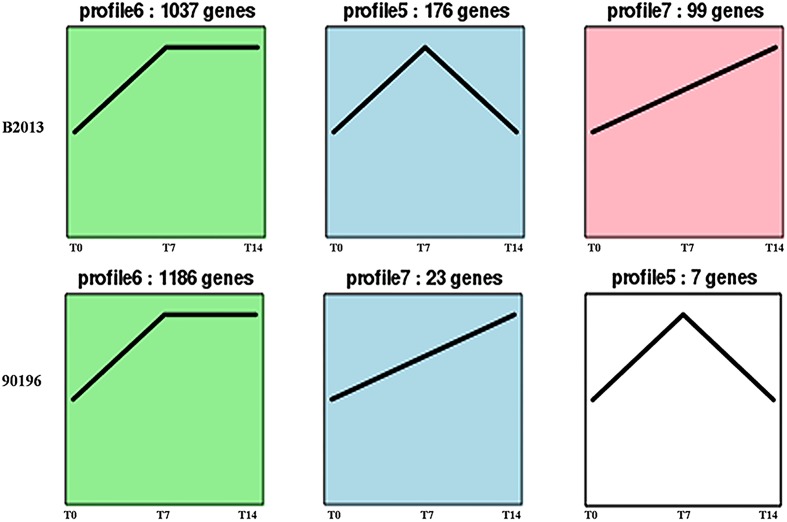
**Patterns of gene expressions across three time points in B2013 and 90196 inferred by STEM analysis (*p* < 0.05).** The black line represented the expression tendency of all the genes. The number of genes belonging to each pattern was labeled above the frame.

The DEGs of B2013 and 90196 from different stages were clustered into eight profiles based on gene expression patterns using STEM software. The profiles showed similar patterns in gene expression over time in response to *P. brassicae* between the two genotypes. As shown in **Figure 3**, most of the DEGs were significantly overrepresented in the profiles exhibiting apparent changes in expression levels at T7 (Profiles 5 and 6, *p* < 0.05), whereas a small percentage of the DEGs were overrepresented in Profile 7 (*p* < 0.05), in which gene expression levels increased over the time course of infection. Next, the DEGs belonging to the overrepresented profiles were subjected to GO-term analysis and KEGG classifications (Supplementary Tables S2 and S3). From the GO category of biological process in B2013, genes involved in cytoskeleton metabolic process, cell wall organization or biogenesis, among other processes, were enriched in Profile 5, in which gene expressions were increased at T7 but decreased at T14. While, just a few GO terms of biological process were notedly enriched in 90196 due to only seven DEGs from this profile. In Profile 6 of both genotypes, the overrepresented GO terms of biological process included DNA repair, response to stimulus and some other processes. The expression level of these genes peaked at T7 and maintained at high level during the subsequent stage. It was worth noting that we found that some DEGs in Profile 6 associated with negative regulation of response to stimulus were uniquely enriched in 90196. In the Profile 7, no DEGs of were enriched in biological process terms.

### Functional Annotation of DEGs at T7

Using a cutoff of a twofold change in gene expression, a total of 2,974 up- and 239 down-regulated genes were identified in B2013 at T7, whereas 2,115 up- and 136 down-regulated genes were found in 90196 at T7. GO enrichment and KEGG pathway analysis of the up-regulated genes revealed some differences between the two genotypes. At T7, a total of 2,415 and 1,790 DEGs were assigned to all three GO categories—biological processes, cell components, and molecular functions—and 68 and 66 GO terms (biological process) were notably enriched in B2013 and 90196, respectively (*q* < 0.05; Supplementary Table S4). Strikingly, some GO terms involved in DNA repair, response to stress and microtubule-based process were enriched at T7 in B2013 (*q* < 0.05). In 90196, some DEGs were also placed in the terms of DNA repair, response to stress, among others. Besides, many helicase-encoding genes were also up-regulated both in B2013 and 90196 at T7.

### Functional Annotation of DEGs at T14

At T14, a total of 2,110 up- and 1,244 down-regulated genes were identified in B2013, and a total of 2797 up- and 1069 down-regulated genes were identified in 90196. GO-term analysis of the up-regulated genes revealed that just a few terms were significantly enriched in B2013, while some enriched GO terms included organic substance metabolic process, primary metabolic process, among others, in 90196 (Supplementary Table S4). Further, down-regulated genes were subjected to GO and KEGG enrichment analysis (Supplementary Tables S4 and S5). DEGs involving in the pathways of phenylpropanoid biosynthesis, plant hormone signal transduction, phenylalanine metabolism, glucosinolate biosynthesis, starch and sucrose metabolism were significantly enriched in two genotypes, which may be participated in response to *P. brassicae* infection. Detailed genes functions will be discussed below (see Defense Responses to *P. brassicae* are Induced Earlier, and Related Pathways are Repressed at T14).

### DEGs Involved in the Response to *P. brassicae* between B2013 and 90196

**FIGURE 4:**
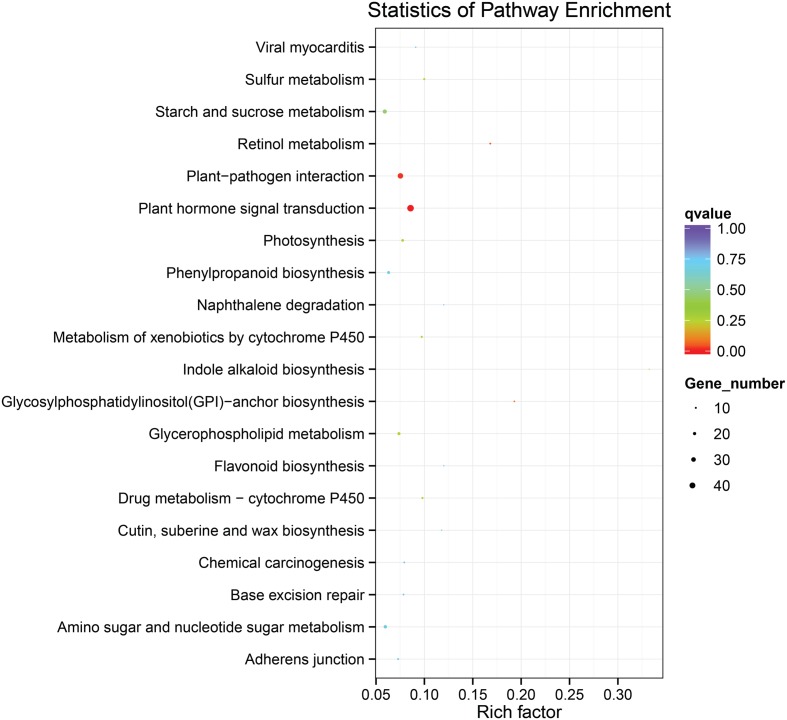
**The top 20 KEGG pathways with the highest representation of common DEGs from pairwise comparisons between the two genotypes**.

**FIGURE 5:**
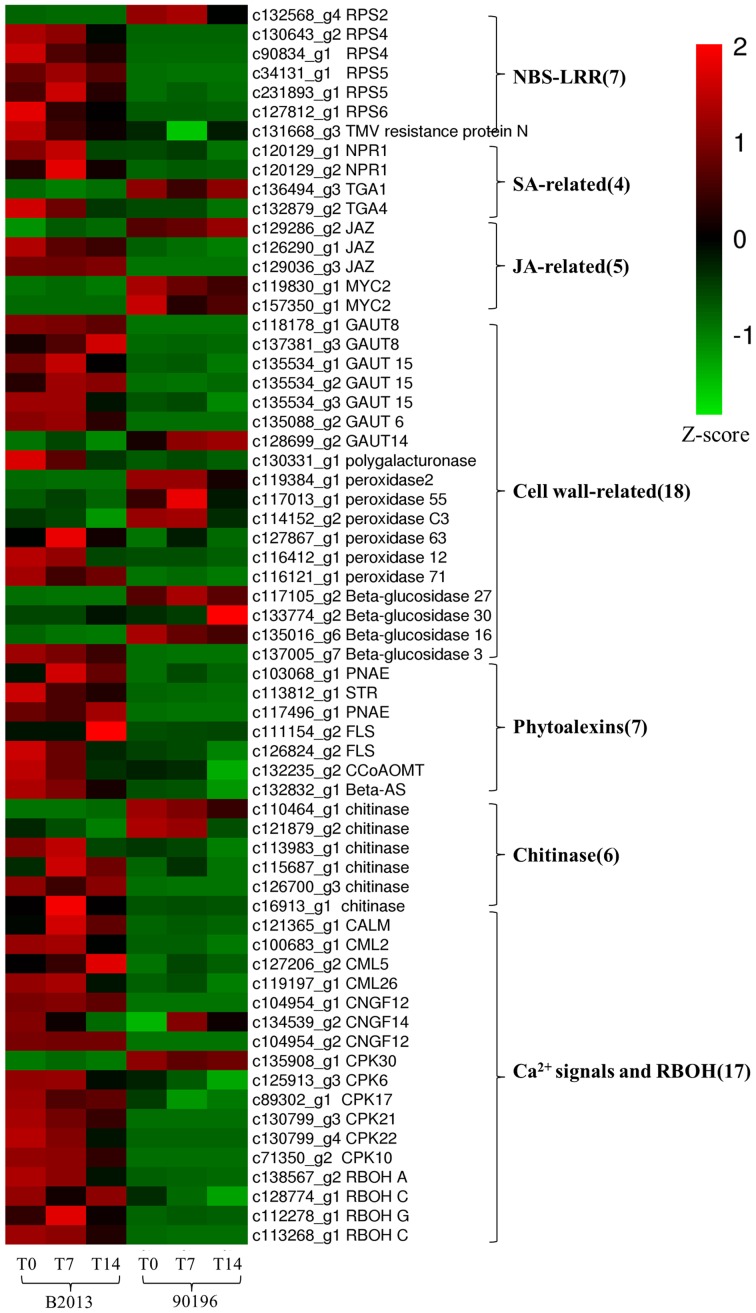
**Heatmap of common DEGs related to defense response to clubroot in B2013 and 90196**.

To identify the genes responsible for the differences in clubroot resistance between B2013 and 90196, pairwise comparisons between the two genotypes were conducted, and 10,532, 10,051, and 6,776 DEGs were identified at T0, T7, and T14, respectively. The large number of DEGs was ascribed to the fact that genotypes from different genetic backgrounds were evaluated in the analysis. Based on these results, we chose 4,516 common DEGs for assignment in KEGG enrichment analysis, regardless of infection stage. The top 20 KEGG pathways with the highest representation of DEGs are shown in **Figure 4**; **Table 3**. Our KEGG pathway enrichment analysis of the DEGs indicated that genes related to pathogen responses were significantly enriched, including “Plant hormone signal transduction,” “Plant–pathogen interaction,” and others. For a global view of the DEGs involved in resistance to *P. brassicae*, the FPKM values for 64 DEGs between two genotypes were represented in a heat map (**Figure 5**) and Supplementary Table S6. The DEGs included seven NBS-LRR containing *R* genes, four SA metabolism related genes, five JA metabolism related genes, eight cell wall biosynthesis or modification related genes, seven phytoalexins, six chitinase, and 17 Ca^2+^ signaling and RBOH related genes. Detailed genes functions will be discussed below (see DEGs Involved in the Response to *P. brassicae* may Contribute to Genotypic Differences in Disease Symptoms).

**Table 3:** Top 20 enriched KEGG pathways with the highest representation of DEGs between B2013 and 90196.

Pathway	Pathway ID	DEGs genes with pathway annotation	Background genes with pathway annotation
Plant hormone signal transduction	ko04075	46	502
Plant–pathogen interaction	ko04626	37	462
Retinol metabolism	ko00830	9	53
Glycosylphosphatidylinositol (GPI)-anchor biosynthesis	ko00563	7	36
Sulfur metabolism	ko00920	11	108
Drug metabolism – cytochrome P450	ko00982	11	110
Metabolism of xenobiotics by cytochrome P450	ko00980	11	111
Glycerophospholipid metabolism	ko00564	19	248
Photosynthesis	ko00195	16	200
Indole alkaloid biosynthesis	ko00901	3	9
Starch and sucrose metabolism	ko00500	27	430
Phenylpropanoid biosynthesis	ko00940	18	274
Cutin, suberine, and wax biosynthesis	ko00073	5	42
Amino sugar and nucleotide sugar metabolism	ko00520	21	335
Adherens junction	ko04520	10	134
Flavonoid biosynthesis	ko00941	4	33

## Discussion

### Difference in Infection Processes for Different Types of Resistance

The clubroot disease presents a widespread and devastating damage to the plants of Brassicaceae family. To date, studies of the molecular basis of clubroot resistance from *Brassica* crops mainly focused on qualitative resistance, especially in Chinese cabbage (Chen et al., 2016) and canola (Chu et al., 2014). However, illuminating molecular mechanism of quantitative resistance could lead to insight into the relationship between qualitative and quantitative resistance, thus to guild utilization of the two types to produce durably resistant cultivar (Jubault et al., 2013). The previous results have showed that the clubroot resistance from the wild relative B2013 was quantitative resistance controlled by multiple genes (Zhang et al., 2014). In our study, transcriptome of broccoli 90196 and its wild relative B2013 was investigated after infection with *P. brassicae* at two developmentally distinct stages. Microscopic observations revealed that root–hair infection (7 DAI) and cortical infection (14 DAI) were both present in B2013 and 90196 (**Figure 1**). This finding was not in agreement with previous studies (Feng et al., 2013; Chen et al., 2016), in which secondary infection did not occur in resistant lines, possibly due to qualitative resistance under single-gene control.

### Plant Cytoskeleton and DNA Repair Act as an Early Response to *P. brassicae*

The plant cytoskeletons play positive roles in defense against invasion and expansion of the pathogen to plant hosts (Takemoto and Hardham, 2004). Plant–pathogen interactions result in reorganization of microtubules and microfilaments toward attempted sites of penetration, restricting pathogens primarily through the deposition of callose and antimicrobial material (Takemoto et al., 2003; Hardham et al., 2007). In current study, some DEGs associated with microtubule-based process were exclusively up-regulated during an earlier stage (T7) in B2013, yet similar transcriptional changes did not observed in 90196 at the same stage, indicating that the microtubule may participated directly in host defense response to *P. brassicae* in resistant genotype. Besides, the genes that were involved in DNA repair participated in an early response to *P. brassicae*, due to detecting many up-regulated helicase-encoding genes both in B2013 and 90196 at T7, corroborating the findings of Zhou et al. (2016) regarding the response to Cd accumulation in *Brassica chinensis*.

### Defense Responses to *P. brassicae* Are Induced Earlier, and Related Pathways are Repressed at T14

#### Cell Wall Biosynthesis

Within the phenylpropanoid biosynthesis pathway (ko00940), 24 genes associated with peroxidase (POD), participating in the biosynthesis of guaiacyl and syringyl lignin, were enriched in B2013 and 90196. Guaiacyl and syringyl are crucial components in the cell wall of angiosperm plants (Obst, 1982). Within the pentose and glucuronate interconversion pathway (ko00040), which is involved in the biosynthesis of cell wall components, eight genes encode the two key enzymes (pectinesterase and polygalacturonase) participating in D-galacturonate biosynthesis, which is essential for forming the backbone of pectic acid (Ridley et al., 2001). The results implied that cell wall biosynthesis might be suppressed by *P. brassicae* infection at T14, consistent with observations obtained from the GO enrichment analysis of expression patterns (Profile 5). Two trehalose 6-phosphate synthase (TPS)-encoding genes were also down-regulated in B2013 at T14, which was observed previously and proposed as a defense mechanism in *A. thaliana* (Gravot et al., 2011).

#### Glucosinolate Biosynthesis

Glucosinolates (GSLs), a group of sulfur-containing plant secondary metabolites found in the Brassicaceae family, have long been postulated to contribute to host–*P. brassicae* interactions (Ludwig-Müller et al., 2009). In the glucosinolate biosynthesis pathway, four cytochrome P450s were found to be down-regulated at T14 compared with T7, among which CYP79B2 and the homolog CYP79B3 catalyze the conversion of tryptophan to indole-3-acetaldoxime (IAOx) (Mikkelsen et al., 2000), and CYP83A1 and CYP83B1 are oxime-metabolizing enzymes (Zhu et al., 2012). Previous studies have found correlations between resistance and a low indole GSL content (Butcher et al., 1974; Ockendon and Buczacki, 1979; Chong et al., 1985), whereas Mullin et al. (1980) were not able to correlate indole GSL and resistance to clubroot. In the present study, the observed down-regulation of four cytochrome P450-encoding genes revealed low-indole GSL levels at T14, which may have contributed to the reduction of disease symptoms, although large clubs did appear in 90196.

#### Plant Hormone Signal Transduction

Plant hormones play pivotal signaling roles in host–*P. brassicae* interactions. In the present study, some genes involved in host auxin biosynthesis as well as auxin transport and response were found to be down-regulated at T14, and these changes occurred in both the susceptible and partially resistant responses. The down-regulation of this pathway was consistent with the reduced gall formation observed in B2013, while large clubs still appeared in 90196. Like the previous findings in *B. napus*, IAA-biosynthesis related genes were also induced by *P. brassicae* infection at the primary stage of the infection process (Xu et al., 2016). In addition, the SA and JA pathways were also repressed due to the down-regulation of related genes at T14. The different roles of SA and JA in defense responses between partial resistance and susceptibility to clubroot will be discussed below.

Overall, the pathways related to the defense response to *P. brassicae* were repressed at T14 in both genotypes. Additionally, most defense genes were up-regulated at T7 compared with T14, indicating that the defense responses to *P. brassicae* were induced quickly.

### DEGs Involved in the Response to *P. brassicae* May Contribute to Genotypic Differences in Disease Symptoms

#### NBS-LRR Proteins May be Involved in Resistance to *P. brassicae* in B2013

Plant NBS-LRR-containing *R* genes can specifically recognize and interact with corresponding pathogen *avr* genes and are considered plant genetic factors that are involved in multiple layers of defense mechanisms (Yu et al., 2014). In the plant–pathogen interaction pathway, five of the six transcripts homologous to *Pseudomonas syringae*-resistant *Arabidopsis* genes were up-regulated in B2013 (one *RPS2*, two *RPS4*, twp *RPS5*, and one *RPS6*), whereas *RPS2* was not. It has been reported that *RPS4*, *RPS5*, and *RPS6* could be involved in resistance to *P. syringae* in *A. thaliana* (Ade et al., 2007; Kim et al., 2009), while *RPS4* and *RPS5* may be involved in the resistance interaction between *P. brassicae* and *B. rapa* (Chen et al., 2016). One gene encoding a TMV resistance protein was also up-regulated in B2013. The enhanced expression of *R* genes in B2013 could represent an activation of a general “surveilance” against pathogen attempts to suppress the PTI response and trigger activation of ETI response and the subsequent defense response, which also suggested that the ETI response was more robust in resistant genotype. These R genes should be used as candidate genes for further investigation.

#### The SA and JA Signal Transduction Pathways Respond Differently in Clubroot-Resistant and Clubroot-Susceptible Genotypes

Extensive studies have shown that SA is involved in the activation of systemic acquired resistance (SAR) in plants such as *A. thaliana*, tobacco and rice (Durrant and Dong, 2004; Vallad and Goodman, 2004; Yuan et al., 2007). Two *NPR1* homologs, identified as key regulators of SA-mediated resistance in *A. thaliana*, were up-regulated in B2013 after inoculation with *P. brassicae*, leading to the activation of downstream SA signaling. TGA factors can interact with NPR1, among which TGA1 and TGA4 are responsible for the SA-dependent interaction (Després et al., 2003). One *TGA4* was up-regulated in B2013, and one *TGA1* was weakly down-regulated in B2013, indicating that the *TGA4*-NPR1 interaction in the SA-dependent pathway may contribute to disease resistance to *P. brassicae* in B2013.

The JAZ (jasmonate-zim domain) proteins act as JA co-receptors and transcriptional repressors in JA signaling in *Arabidopsis* (Kazan and Manners, 2012), interacting with the positive regulator of JA signaling MYC2 (Chini et al., 2007). Thus, MYC2 inactivation results in impaired JA signaling. Two of three genes encoding JAZ proteins were up-regulated in B2013, and *MYC2* was up-regulated in 90196. The results showed that the JA pathway was weakly activated in the resistant genotype but strongly induced in the susceptible genotype, in agreement with results reported for *Arabidopsis* by Lemarié et al. (2015). Chen et al. (2016) also demonstrated that the SA signaling pathway, but not the JA/ET signaling pathway, played a critical role in *B. rapa* resistance to *P. brassicae* infection. Jubault et al. (2013) also found that both the SA and ET pathways were induced during the partial resistance response, whereas the JA pathway was repressed.

#### Enhanced Cell Wall Biosynthesis has a Link to Resistance to *P. brassicae*

In terms of starch and sucrose metabolism, many genes involved in the biosynthesis of cell wall components exhibited different expression levels, suggesting that different cell wall-related functions were involved in response to *P. brassicae* in the two genotypes. Seven of 27 over-expressed genes within this pathway encode the key GAUT (α-1,4-galacturonosyltransferase) enzymes, which are essential for the biosynthesis of the plant cell wall component pectic polysaccharide homogalacturonan (HGA) (Sterling et al., 2001). With the exception of *GAUT14*, the other *GAUT* genes (one *GAUT6*, two *GAUT8*, and three *GAUT15*) were up-regulated in B2013 compared with 90196 at three stages. The key enzyme polygalacturonase was also elevated in B2013 at these stages. In another phenylpropanoid biosynthesis pathway, six genes associated with POD that participate in the biosynthesis of guaiacyl and syringyl lignin were up-regulated in B2013. Similar increases in lignin synthesis in roots have been observed in Cd-stressed pakchoi (Zhou et al., 2016) and *Verticillium dahliae*-inoculated cotton (Xu et al., 2011). This study demonstrates that lignification and reinforcement of cell walls are important processes in the response of plants to *P. brassicae* penetration.

Interestingly, three of four enzymes (β-glucosidase) involved in the hydrolysis of cellulose were up-regulated in 90196 compared with B2013, and one was up-regulated in B2013. We inferred that host cell wall components may be degraded by hydrolytic enzymes during *P. brassicae* infection through unknown pathways, furthering the pathogen infection process. The activity of hydrolytic enzymes was higher in the susceptible genotype, resulting in a severe infection and symptoms.

#### Phytoalexins May Contribute to Resistance toward *P. brassicae* in Resistant Genotypes

Resistant species can also be colonized by the fungus, followed by a severe basal defense response and rapid induction of phytoalexin production. Phytoalexins are defined as “any low-molecular-weight, anti-microbial secondary metabolites that are synthesized and accumulate in plants in response to biotic and abiotic stress” (Ahuja et al., 2012). Phytoalexins have been characterized amongst different classes of chemical compounds such as coumarins, terpenoids, flavonoid, alkaloids, stilbenes, phenolic compounds, and many others (Arruda et al., 2016). According to our pathway enrichment analysis, the indole alkaloid biosynthesis, flavonoid biosynthesis, and triterpene biosynthesis pathways exhibited more up-regulated genes in resistant lines than in susceptible lines.

Indole alkaloids are tryptophan-derived alkaloids that are important secondary metabolites and can protect plants against adverse environments, and particularly microbe invasion or insect grazing (Miranda-Ham et al., 2007). Three genes encoding the two key enzymes (two polyneuridine-aldehyde esterase [PNAE] and one strictosidine synthase [STR]) involved in indole alkaloid biosynthesis showed 2.7- to 6.3-fold higher expression in B2013 than in 90196. These genes reached their highest expression level at T14, suggesting that B2013 may have a higher indole alkaloid content, as found in aphid-resistant wheat leaves (Cai et al., 2004).

In the current study, four genes related to flavonoid biosynthesis were identified, including two up-regulated *flavonol synthase (FLS)* genes and one up-regulated *caffeoyl-CoA O-methyltransferase (CCoAOMT)* gene. *FLS*, which catalyzes the reaction from dihydroflavonol to flavonol, is the key enzyme for flavonol biosynthesis (Holton et al., 1993). Flavonol plays a role as a UV protectant (Flint et al., 1985) and in scavenging ROS (Vu et al., 2015). In *A. thaliana*, transcripts of selected genes involved in each step of flavonoid biosynthesis are up-regulated during clubroot development (Ludwig-Müller et al., 2009). CCoAOMT activity in cultured carrot cells was found to be rapidly induced in response to fungal elicitors (Kühnl et al., 1989). Recently, *CCoAOMT* has been shown to participate in lignin biosynthesis in woody poplar (Zhong et al., 2000), and down-regulation of *CCoAOMT* via RNA interference leads to reduced lignin production in the cell wall of maize straw (Li et al., 2013). We deduced that up-regulation of *CCoAOMT* may lead to an increased lignin content, thickening the cell wall of the host and reducing root tissue damage due to *P. brassicae* infection in B2013.

Triterpenes, a major subgroup of the terpene superfamily, are valuable metabolites with antiviral and antibacterial properties (Alakurtti et al., 2006) that are derived from the cyclization of 2, 3-oxidosqualene by oxidosqualene cyclases (OSCs) (Delis et al., 2011). The β-AS (β-amyrin synthase) gene, encoded by *OSC3*, has been shown to be required for the synthesis of defense compounds (Haralampidis et al., 2001). In the present study, one β-AS-related gene was found to be up-regulated in B2013 compared to 90196.

In summary, phytoalexins including indole alkaloids, indole alkaloids, and triterpenes were shown to act as defense compounds against *P. brassicae*. Seven genes responsible for the biosynthesis of different types of phytoalexins were markedly up-regulated in B2013 at various stages compared with 90196 and were therefore thought to likely contribute to resistance to *P. brassicae* in resistant genotype. However, few genes involved in phytoalexins biosynthesis were found to be differentially expressed between different resistance in *Arabidopsis* (Jubault et al., 2013).

#### Chitinase Responds Differently to *P. brassicae* Infection in Resistant and Susceptible Genotypes

We identified six chitinase genes that were differentially expressed between the two genotypes, among which four were up-regulated in B2013. Chitinases catalyze the hydrolysis of chitin, a major component of the cell walls of *P. brassicae* (Moxham and Buczacki, 1983), and are a type of pathogenesis-related (PR)-like protein that has been used to trigger defense responses against fungal pathogens (Xian et al., 2012). There is still no direct evidence demonstrating that chitin released by *P. brassicae* is PR, although several chitin synthases are highly expressed in *P. brassicae* during infection (Schwelm et al., 2015). Four up-regulated genes showed 1.7- to 8.9-fold higher expression in B2013 than in 90196, reaching the highest expression level at T14, revealing that the activity of chitinase increased gradually as infection progressed, while two down-regulated genes reached their highest expression level at T0 (c110464_g1) or T14 (c121879_g2). These results showed that different chitinases played distinct roles at different infection stages, as observed in *B. rapa* by Chen et al. (2016). The chitinase genes that showed higher expression levels during secondary infection appeared to likely be more conducive to triggering host defenses against *P. brassicae* in the current study.

#### Ca^2+^ Signaling may be Involved in the Response to *P. brassicae* Infection by Activating RBOH Proteins

Genes related to Ca^2+^ influx have been suggested to play a key role in the genotypic differences underlying clubroot resistance (Chen et al., 2016). Changes in cytosolic Ca^2+^ levels are a primary response to biotic and abiotic stress (Rudd and Franklin-Tong, 2001). Three genes (two *CNGC12*, one *CNGC 14*) encoding cyclic nucleotide gated channels (CNGCs) were markedly up-regulated in B2013 compared with 90196 at three stages. CNGCs are a group of cation channels that mediate Ca^2+^ influx into the cytosol following activation via ligand binding (Jammes et al., 2011). They play important roles in the response to signals from pathogens and pathogen-associated molecular pattern (PAMP) signals (Ma, 2011). Calmodulins (CaMs) and calmodulin-like proteins (CMLs) are also involved in the pathogen response signaling cascade, functioning as Ca^2+^ sensors (Ma, 2011). The genes related to CaM, CML2, CML5, and CML26 were all up-regulated in B2013 compared with 90196. In addition, four of six calcium-dependent protein kinase (CPK) genes (*CPK 10, 17, 21, 22*) were also up-regulated in B2013 at three time points. *CPK*, a family of serine/threonine protein kinases that are unique to plants and some protists, may be involved in multiple signal transduction pathways (Martín and Busconi, 2001). Moreover, Ca^2+^ signals can activate respiratory burst oxidase homolog (RBOH) proteins, which are involved in mediating the production of reactive oxygen species (ROS) (Kurusu et al., 2015). As expected, four RBOH-related genes (one *RBOHA*, two *RBOHC*, one *RBOHG*) were up-regulated in B2013, indicating that a higher ROS level in the resistant genotype contributed to inhibition of colonization by *P. brassicae* in the roots, in agreement with previous findings in *B. rapa* after infection by *P. brassicae* (Chen et al., 2016).

## Conclusion

We first investigated the transcriptome response in the roots of clubroot -susceptible broccoli line and its clubroot-resistant wild relative line during the different stages of *P. brassicae* infection. In our study, two findings that (1) the response changes in transcript level of two genotypes under *P. brassicae* infection were mainly activated at the primary stage (T7). Some pathways related to cell wall and glucosinolate biosynthesis and plant hormone signal transduction were repressed at T14(the secondary stage) compared to T7, and (2) the pathways associated with response defense to clubroot were activated in the resistant genotype. Our results could provide new insights into the molecular mechanisms underlying the resistance to *P. brassicae.*

## Author Contributions

XZ performed the experiments, analyzed the data, wrote and revised the manuscript; YL designed the research and critically edited the manuscript; ZF guided the experimental design; ZL and LY performed the data analysis; and MZ, YZ, and HL planted and managed the plants. All authors approved the final manuscript.

## Conflict of Interest Statement

The authors declare that the research was conducted in the absence of any commercial or financial relationships that could be construed as a potential conflict of interest.

## Footnotes

1 http://www.ncbi.nlm.nih.gov/COG/

2 http://pfam.sanger.ac.uk/

3 http://www.ebi.ac.uk/uniprot/

4 http://www.genome.jp/kegg/

5 http://www.geneontology.org/

6 http://www.premierbiosoft.com/crm/jsp/com/pbi/crm/clientside/ProductList.jsp

## Supplementary Material

FIGURE S1 **Disease symptoms of 90196 (A)** and B2013 **(B)** 42 day after *P. brassicae* inoculation.
