# Supplementary figures and images for: Comparative Transcriptome Analysis between Broccoli (Brassica oleracea var. italica) and Wild Cabbage (Brassica macrocarpa Guss.) in Response to Plasmodiophora brassicae during Different Infection Stages
Source: Front Plant Sci. 2016 Dec 23;7:1929. doi: 10.3389/fpls.2016.01929 (PMC5179516; doi:10.3389/fpls.2016.01929)

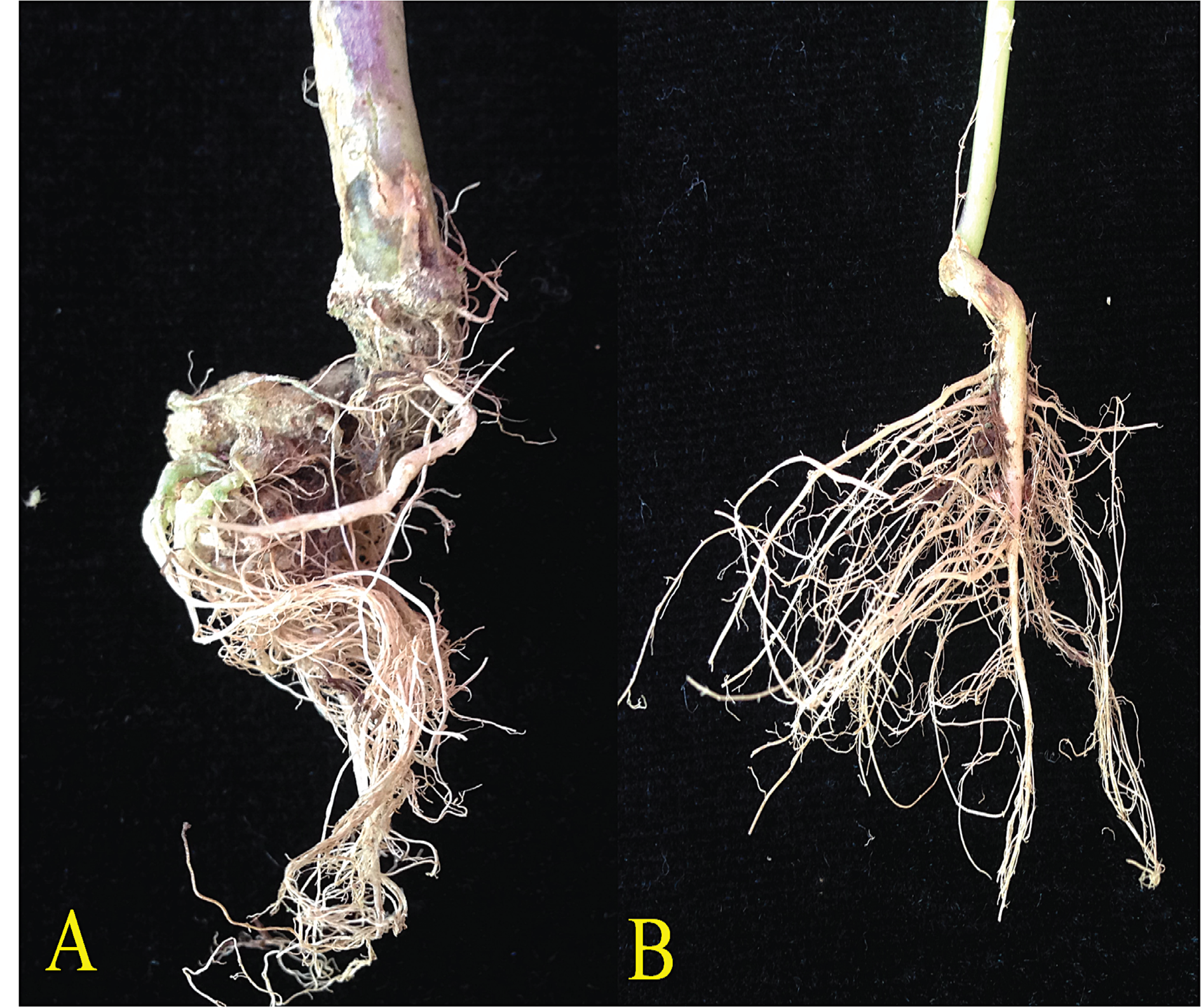

Supplement: FIGURE S1 — Disease symptoms of 90196 (A) and B2013 (B) 42 day after P. brassicae inoculation. [file Image_1.TIF]
